# Pharmacokinetic Comparisons of Eight Active Components from Raw Farfarae Flos and Honey-Processed Farfarae Flos after Oral Administration in Rats by UHPLC-MS/MS Approaches

**DOI:** 10.1155/2020/4091816

**Published:** 2020-05-20

**Authors:** Liu Yang, Hai Jiang, Xinyue Guo, Ajiao Hou, Wenjing Man, Song Wang, Jiaxu Zhang, Bingyou Yang, Jianmin Li, Haixue Kuang

**Affiliations:** ^1^Heilongjiang University of Chinese Medicine, Harbin 150040, China; ^2^First Affiliated Hospital of Heilongjiang University of Chinese Medicine, Harbin, China

## Abstract

Farfarae flos (FF) is widely used for cough over thousands of years in China, but little is known about their pharmacokinetics properties. This study was aimed to establish a rapid and accurate ultraperformance liquid chromatography with triple-quadrupole tandem mass spectrometry method for compare pharmacokinetics studies of eight active compounds after oral administration between raw and honey-processed farfarae flos extracts. Optimum separation was performed on a Thermo Hypersil GOLD C18 column (100 mm × 2.1 mm, 1.9 *µ*m particles size) with a gradient elution of acetonitrile as mobile phase A and 0.3% formic acid aqueous solution as mobile phase B. The flow rate was set as 0.3 mL/min and separated for 34.0 minutes. Electrospray ionization in the negative ion mode and selected reaction monitoring were used to identify and separate active components. The results met the acceptance criteria and showed that this method exhibited good linear, precision, accuracy, and stability. The extraction recoveries ranged from 81.54% to 104.48%, and the matrix effects ranged from 81.94% to 103.02%. These results show that the validated method could be successfully applied to evaluate the pharmacokinetic study in rats after oral administration of raw farfarae flos (R-FF) and honey-processed farfarae flos (H-FF).

## 1. Introduction

Generally, before traditional Chinese medicine (TCM) is used for their specified clinical application, its raw herbs are commonly specially processed, which is called “paozhi” in Chinese [1]. Traditional Chinese medicine processing methods are wine-processed, salt-processed, vinegar-processed, honey-processed, Glycyrrhizae Radix et Rhizoma-processed, sulfur fumigation, and so on [2, 3]. The purpose of TCM processing is to promote therapeutic effects, eliminate toxicity, reduce side effects, and change the properties of raw herbs.

Farfarae flos (FF) belong to the Compositae family, the dried flower buds of Tussilago farfara L., is one of the widely used TCM over thousands of years, and it is often employed for the treatment for cough, expectorant, acute and chronic tracheitis, and asthma [4–6]. As was recorded in “Ten Effective Remedies”, honey-processed farfarae flos (H-FF) can enhance the effect of moistening lung for arresting cough, indicating that honey-processed FF enhances the FF clinical effect. It is important that H-FF is the best choice of cough medicine, which has a long history been widely applied in many Chinese patent medicines such as Zhike Juhong Koufuye and Zhisou Qingguo Heji [7, 8]. Even though FF have a long history of clinical application, little is known about their pharmacokinetics properties. Thus, the dynamics of raw and H-FF in vivo are not well understood on account of absence of scientific evidence and research method.

Much attention had been drawn to FF due to significant pharmacological effects, such as antiplatelet aggregation, anti-inflammatory, antiallergic, antitumor, and other effects [9]. FF has complex chemical ingredients containing volatile oils, phenolic acids, flavonoids, polysaccharides, sterols, alkaloids, and terpenes. But it draws attention that modern phytochemical studies have suggested that the part of total phenolic acids of FF has moistening lung for arresting cough activities [6]. And, for all we know, the total phenolic acid contained in FF mainly consists of chlorogenic acid, cryptochlorogenic acid, neochlorogenic acid, isochlorogenic acid B, isochlorogenic acid C, caffeic acid, and ferulic acid [5, 10]. Moreover, rutin has obvious pharmacological effects such as anti-inflammatory, antioxidant, reducing blood fat, and certain protective effects on nerves [11]. We suspect that the clinical efficacies of the H-FF are different from raw, which may be on account of bioactive compounds' pharmacokinetics changes in vivo. However, previous research on FF and its processed products have often focused on the quantitative analysis [12] and change of pharmacological actions [6], but the pharmacokinetics differences of bioactive compounds after the oral administration of R-FF and H-FF extracts had never been studied yet. To understand the dynamic changes of active compounds in vivo, it is essential to develop a precise and rapid method to compare these active compounds' pharmacokinetics difference of raw and H-FF.

In this study, a precise and rapid UHPLC-MS/MS approach was first established for simultaneous determination of chlorogenic acid, cryptochlorogenic acid, neochlorogenic acid, isochlorogenic acid B, isochlorogenic acid C, caffeic acid, ferulic acid, and rutin in rat serum. It is also the first study applied to the comparative pharmacokinetics of raw and its honey-processed products. At the same time, the effect of honey-processed FF on pharmacokinetic behaviors of FF active compounds was elucidated.

## 2. Experimental

### 2.1. Chemicals

**Figure 1:**
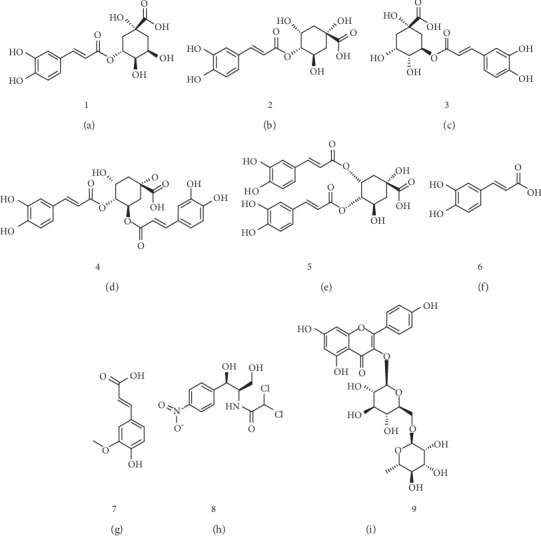
Compound structures of (a) chlorogenic acid, (b) cryptochlorogenic acid, (c) neochlorogenic acid, (d) isochlorogenic acid B, (e) isochlorogenic acid C, (f) caffeic acid, (g) ferulic acid, (h) rutin, and (i) chloramphenicol (IS).

Authentic standards of chlorogenic acid, cryptochlorogenic acid, neochlorogenic acid, isochlorogenic acid B, isochlorogenic acid C, caffeic acid, ferulic acid, rutin, and chloramphenicol (internal standard, IS) were purchased from Chengdu Must Biotechnology (Chengdu, China). The purity of these standards was determined more than 98% by ultraperformance liquid chromatography/photo diode array (UPLC-PDA) detector. The structures of these standards are shown in Figure 1. HPLC grade methanol and HPLC grade acetonitrile were bought from Fisher Scientific (Janssen Pharmaceuticalaan 3a 1 Reagent Lane). HPLC grade formic acid (FA) was obtained from Dikma Co. (USA). The purified water was bought from Wahaha (Hangzhou, China). All other chemical reagents are of analytical grade.

R-FF and H-FF were purchased from Harbin Chinese herbal medicine market. All samples were identified as the FF (Compositae) by Professor Su Lianjie from Heilongjiang University of Chinese Medicine, Harbin, China. All samples were dried at 25°C and kept at a constant temperature storage.

### 2.2. Chromatographic and Mass Spectrometric Conditions

MS analysis was performed on a TSQ Quantis^™^ triple quadrupole mass spectrometer (Thermo Scientific^™^, Vanquish^™^, (Waltham, MA, USA)) equipped with an autosampler. Chromatographic separation was performed on a Thermo Hypersil GOLD C18 column (100 mm × 2.1 mm, 1.9 *µ*m particles). The column temperature was maintained at 30°C. Through the Thermo Scientific™, TraceFinder™ software was acquired and processed. The mobile phase was composed of acetonitrile (A) and water (both containing 0.3% formic acid) (B) with a gradient elution program (0–5 min:10–19% acetonitrile, 5–8 min:19–25% acetonitrile, 8–28 min:25%–100% acetonitrile, 28–32 min: 100%–10%, and 32–34 min: 10%–10%). The system was re-equilibrated in the initial mobile phase 4.0 min before the next injection. The flow rate was set at 0.3 mL/min, and the injection volume was set as 2 *μ*L.

The analytes were detected on a TSQ Quantis triple quadrupole mass spectrometer by selected reaction monitoring (SRM) equipped with a heated electrospray ionization source operated in negative ion modes. The parameters were set as follows: the sheath gas and the aux gas were set at 30 Arb and 10 Arb, respectively; the ion transfer tube temperature was maintained at 325°C; and the vaporizer temperature was set to 350°C.

### 2.3. Preparation of Extraction of R-FF and H-FF

Aliquots (70 g) of R-FF and H-FF were individually boiled in 85% ethanol 3 times for 1 h each time, and then it was filtered through gauze, respectively. The extracts were then merged by evaporation and concentration of rotary evaporators. Finally, R-FF and H-FF extracts were rediluted to the same concentration by UHPLC-MS/MS and quantitatively determined using the same chromatography conditions as described above, respectively. The corresponding concentrations of chlorogenic acid, cryptochlorogenic acid, neochlorogenic acid, isochlorogenic acid B, isochlorogenic acid C, caffeic acid, ferulic acid, rutin, and were 4557.54, 622.01, 483.18, 4920.11, 2895.64, 368.36, 34.63, and 9457.26 *μ*g/g in the R-FF extract and 5396.67, 654.35, 492.09, 2729.93, 1472.28, 598.71, 57.38, and 9612.12 in the H-FF extract, respectively.

### 2.4. Preparation of Standards

Stock solutions (1 mg/mL) of chlorogenic acid, cryptochlorogenic acid, neochlorogenic acid, isochlorogenic acid B, isochlorogenic acid C, caffeic acid, ferulic acid, rutin, and IS were prepared in methanol. Then, the stock solutions were then mixed and serially diluted with methanol-water (50 : 50, v/v) to obtain mixed calibration standards. All solutions were stored at 4°C before each experiment.

### 2.5. Preparation of Calibration Curves

The standard stock solutions and quality control (QC) samples were prepared by spiking 100 *μ*L different concentrations of the mixed calibration standard solutions and 695 *μ*L of acetonitrile into 100 *μ*L blank serum and 5 *μ*L IS. The final serum-derived working solutions of chlorogenic acid, cryptochlorogenic acid, neochlorogenic acid, isochlorogenic acid B, isochlorogenic acid C, caffeic acid, ferulic acid, hyperoside, and rutin were in the range of 4.35–118500.00 ng/mL, 2.38–168000.00 ng/mL, 2.67–39000.00 ng/mL, 1.30–211000.00 ng/mL, 4.87–230000.00 ng/mL, 2.78–8840.00 ng/mL, 2.05–1000.00 ng/mL, and 1.45–8150.00 ng/mL, respectively. The low, middle, and high concentrations of QC samples were prepared containing 3500.00, 40400.00, and 110000.00 ng/mL for chlorogenic acid; 2000.00, 50000.00, and 100000.00 ng/mL for cryptochlorogenic acid; 1000.00, 15000.00, and 25000.00 for neochlorogenic acid; 2000.00, 80000.00, and 160000.00 ng/mL for isochlorogenic acid B; 1500.00, 7500.00, and 140000.00 ng/mL for isochlorogenic acid C; 380.00, 3800.00, and 6000.00 ng/mL for caffeic acid; 100.00, 200.00, and 500 ng/mL for ferulic acid; and 600.00, 4000.00, and 8000.00 ng/mL for rutin, respectively.

### 2.6. Preparation of Serum Samples

5 *μ*L of IS solution containing chloramphenicol 100 ng/ml and 795 *μ*L of acetonitrile were added to a 100 *μ*L drug serum sample for sedimentation of proteins. Subsequently, the 1.5 mL centrifuge tube containing mixture was vortexed for 5 min. After that, the mixture was centrifuged at 12,000 rpm for 20 min at 4°C. Finally, the supernatant fluid was removed and evaporated to dryness under a stream of nitrogen at 35°C. The dried residue was redissolved by 100 *μ*L 50% methanol aqueous solution. And the supernatant prepared above was filtered through 0.22 *μ*m membranes transferred to vials by the UHPLC-MS/MS system for data analysis.

### 2.7. Method Validation

**Figure 2:**
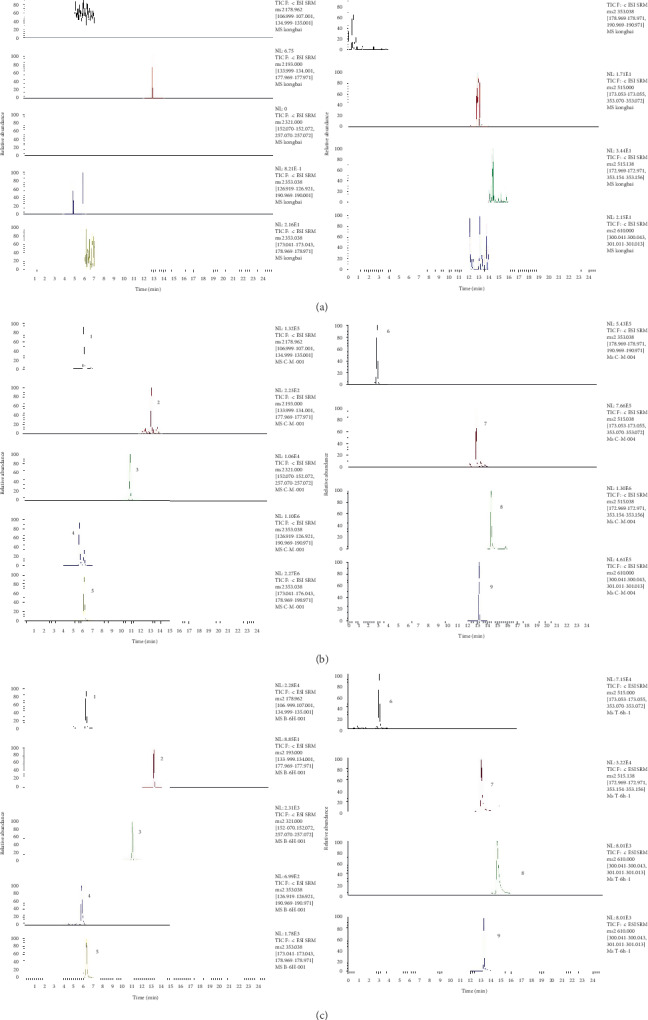
SRM chromatograms of (1) caffeic acid; (2) ferulic acid; (3) chloramphenicol (IS); (4) chlorogenic acid; (5) cryptochlorogenic acid; (6) neochlorogenic acid; (7) isochlorogenic acid B; (8) isochlorogenic acid C; (9) rutin: (a) blank rat sample, (b) plasma spiked with 8 analytes and IS, and (c) plasma sample obtained from a rat 4 h after oral administration.

The specificity of the method was assessed by analyzing the chromatograms of blank serum from different rat plasma samples; a spiked blank serum with mixed calibration standards and IS, and rat serum samples were collected after 4 h oral administration of R-FF extracts which are shown in Figure 2. All samples were observed no endogenous substances interferences at the retention time of the analyte and the IS. The results showed that this method has good selectivity.

The calibration standard curve was prepared by calculating the peak area ratios of the eleven analytes and the IS versus the concentration of respective analytes in plasma with a weighted (1/*X*^2^) linear least-squares regression. Lower limit of quantification (LLOQ) was defined as the analytical concentration of at which the signal-to-noise (S/N) ratio of 10 : 1.

The precision and accuracy were evaluated by six replicates of each concentration of QC samples at three concentration levels on the same day (intraday precision) and consecutive three days (interday precision). Accuracy (100%) was calculated as (measured average concentration−theoretical average− concentration/theoretical average concentration) × 100%. Precision and accuracy were expressed as the relative standard deviation (RSD) and relative error (RE), respectively.

Recovery was calculated by comparing analytes/IS peak area ratios of extracted analytes with that blank serum spiked with pure reference standards. The matrix effect was measured by comparison of the components/IS peak area ratios in the postextraction spiked samples with that acquired from the standard solutions. Both assessments RSD should be less than 15%.

Short-term stability was calculated by keeping the QC samples at room temperature for 4 hours. Long-term stability was investigated by storing the same QC samples at −20°C in a refrigerator for 30 days. The freeze and thaw stability was determined by analyzing the same QC samples after 3 consecutive freeze-thaw (−20 to 20°C) cycles. The postpreparative stability was evaluated by keeping the QC samples at autosampler for 24 hours.

### 2.8. Pharmacokinetic Study

12 adult male SD rats (weighing 250∼300 g) were used in pharmacokinetics experiments and randomly assigned to two groups: R-FF group and H-FF group. Before being administered R-FF or H-FF extract, all the rats fasted but with free access to water for 12 h. The R-FF or H-FF extract was orally administered to the rats (140 g/kg body weight). Aliquots of 0.3 ml blood samples were collected in 1.5 ml heparin lithium-anticoagulant tubes at (0.083, 0.166, 0.333, 0.5, 1, 2, 4, 6, 8, 12, 24, 36, and 48 h) via the postorbital venous plexus veins from each rat after oral administration. The blood sample was immediately centrifuged at 3500 rpm for 5 min, and the supernatant 100 *μ*L serum was transferred into a new centrifuge tube and stored at −80°C until analysis.

## 3. Results and Discussion

### 3.1. Optimization of UHPLC-MS/MS Conditions

By a syringe pump (200 ng/mL analytes and IS were diluted with methanol-water (50 : 50, v/v)) to optimize mass spectrum parameters. The results show that all analytes and IS had higher signal in the negative ionization mode. The SRM modes were analyzed of the precursor ion of chlorogenic acid, cryptochlorogenic acid, neochlorogenic acid, isochlorogenic acid B, isochlorogenic acid C, caffeic acid, ferulic acid, rutin, and IS (m/*z* 353.04, 353.04, 353.04, 515.00, 515.10, 178.96, 193.00, 610.00, and 321.00) were stable in high abundance. The results were showed in Table 1. In order to obtain suitable retention times and good peak shapes, we optimized the chromatographic column, mobile phase, modifier, and gradient elution procedure. In this preliminary experiment, we first chose the ACQUITY UPLC 1.8 *μ*m HSS T3 column (Waters) to carry out elution, but we found partial peak trailing. Then, we chose 1.9 *μ*m Hypersil GOLD column (Thermo) to carry out elution, and we found that all compounds could rapidly and complete separation. When methanol/acetonitrile (mobile phase A) was tested initially as the organic mobile phase, acetonitrile having a higher signal was observed. And as the mobile phase B, containing 0.1% formic acid aqueous solution, 0.2% formic acid aqueous solution, or 0.3% formic acid aqueous solution, increases, we found that 0.3% formic acid can obtain best ionization. Finally, the 1.9 *μ*m Hypersil GOLD column (Thermo) was selected for gradient elution for 34.0 min, and the mobile phase was acetonitrile-water containing 0.3% formic acid at a flow rate of 0.3 mL/min.

### 3.2. Validation of the Method

This developed method was validated by specificity, lower limit of detection (LLOD), lower limit of quantitation (LLOQ), intraday precision, interday precision, accuracy, stability, recovery, recovery, and matrix effect.

The chromatograms of blank serum, blank serum containing mixed standards and IS, and after 4 h oral administration of R-FF extract serum samples are shown in Figure 2. No obvious endogenous interference peak was found at the retention time of both the compounds and the IS. The retention times of chlorogenic acid (5.65 min), cryptochlorogenic acid (6.14 min), neochlorogenic acid (2.94 min), isochlorogenic acid B (12.86 min), isochlorogenic acid C (12.86 min), caffeic acid (6.11 min), ferulic acid (6.11 min), rutin (13.17 min), and IS (10.85 min), respectively.

The linear regressions of chlorogenic acid, cryptochlorogenic acid, neochlorogenic acid, isochlorogenic acid B, isochlorogenic acid C, caffeic acid, ferulic acid, and rutin exhibited good linear relationships in the rat serum. The validated method showed excellent linearity (*r*^2^ > 0.990) in Table 2. The LLOQ of chlorogenic acid, cryptochlorogenic acid, neochlorogenic acid, isochlorogenic acid B, isochlorogenic acid C, caffeic acid, ferulic acid, and rutin was determined as 4.35, 2.38, 2.67, 1.30, 4.87, 2.78, 2.05, 1.45 ng/mL, respectively.

The intraday and interday precisions (RSD%) were calculated at three QC levels, and the result was lower than 4.94%. Also, the result of all compounds accuracies (RE%) ranged from −5.11–4.81%. All results were illustrated in Table 3. The result shows that precision and accuracy were satisfied.

The extraction recoveries of these compounds were found to be satisfactorily ranged from 81.54–104.48% with SD less than 7.60%. The recovery results were satisfied. The matrix effects derived from three concentration QC samples were between 81.94 and101.71% with SD below 12.51%. All results are summarized in Table 4.

Short-term stability, long-term stability, and freeze/thaw stability were tested. These data are summarized in Table 5 and demonstrated that this method was good for the simultaneous determination of chlorogenic acid, cryptochlorogenic acid, neochlorogenic acid, isochlorogenic acid B, isochlorogenic acid C, caffeic acid, ferulic acid, and rutin. The range of variation for all analytes was within 15% of the actual value.

### 3.3. Pharmacokinetic Study

**Figure 3:**
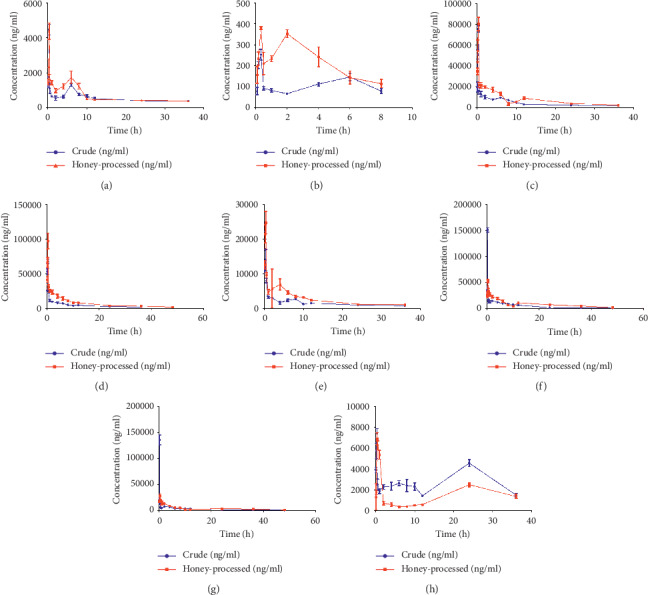
Mean plasma concentration time curves of caffeic acid (a) ferulic acid, (b) chlorogenic acid, (c) cryptochlorogenic acid, (d) neochlorogenic acid, (e) isochlorogenic acid B, (f) isochlorogenic acid C, and (g) rutin, (h) after oral administration of R-FF and H-FF extracts.

To date, much attention had been drawn to the study of pharmacokinetics of TCM, which mainly focused on the study of pharmacokinetics of raw herb, and there are few studies on the pharmacokinetics of processed herb. In our study, the established UHPLC-MS/MS method was sensitive enough for simultaneous determination of chlorogenic acid, cryptochlorogenic acid, neochlorogenic acid, isochlorogenic acid B, isochlorogenic acid C, caffeic acid, ferulic acid and rutin after oral administration R-FF, H-FF extracts. The corresponding concentrations of 8 compounds were 4557.54 *μ*g/g and 5396.67 *μ*g/g for chlorogenic acid, 622.01 *μ*g/g and 654.35 *μ*g/g for cryptochlorogenic acid, 483.18 *μ*g/g and 492.09 *μ*g/g for neochlorogenic acid, 4920.11 *μ*g/g and 2729.93 *μ*g/g for isochlorogenic acid B, 2895.64 *μ*g/g and 1472.28 *μ*g/g for isochlorogenic acid C, 368.36 *μ*g/g and 598.71 *μ*g/g for caffeic acid, 34.63 *μ*g/g and 57.38 *μ*g/g for ferulic acid, and 9457.26 *μ*g/g and 9612.12 *μ*g/g for rutin in R-FF and H-FF extracts, respectively. The mean serum concentration-time curves of the 8 compounds in the two treatments are illustrated in Figure 3. The pharmacokinetic parameters of R-FF and H-FF including *T*_max_, *C*_max_, AUC0–*t*, AUC0–∞, *t*1/2, and MRT are summarized in Tables 6 and 7.

As shown in Table 6, after oral administration R-FF, all compounds could be detected in serum within 5 minutes, and this phenomenon shows that all compounds could be quickly absorbed into the blood circulatory system. It is interesting to note that, after oral administration, all compounds reach the maximum serum concentration in the same 20 min, indicating all compounds had a fast absorption rate. The R-FF group including chlorogenic acid, cryptochlorogenic acid, neochlorogenic acid, isochlorogenic acid B, isochlorogenic acid C, caffeic acid, and rutin pharmacokinetics parameters of the *t*1/2 and the MRT values were relatively long, indicating that they had a slow eliminate rate. While ferulic acid pharmacokinetics parameters of the *t*1/2 and the MRT values were short, relatively speaking they had a fast eliminate rate. In Figure 3, caffeic acid, ferulic acid, neochlorogenic acid, and rutin can observe obvious double peak phenomena both R-FF and H-FF, which are probably due to reabsorption, distribution, and enterohepatic circulation.

Compared to the R-FF (Table 6) group, the honey-processed FF has nearly no effect on the pharmacokinetic parameters of the *T*_max_ values, which revealed that it did not change the rate of absorb of these active compounds. However, there are considerable differences in the other pharmacokinetic parameters of eight compounds investigated between R-FF group and H-FF groups. In the H-FF group, *T*1/2 (h) and MRT0–*t* (h) values of isochlorogenic acid B, isochlorogenic acid C, and rutin were increased, suggesting that processing can slow these compounds elimination rate. We also noticed the *t*1/2 values of chlorogenic acid, cryptochlorogenic acid, and neochlorogenic acid in the H-FF group are a little shorter than the R-FF group, but MRT0–*t* was extended. It shows that the elimination of chlorogenic acid, cryptochlorogenic acid, and neochlorogenic acid in rat's serum has slow rate, which it might be processed can promote the complex chemical matrix transformation affect its metabolism. Interestingly enough, in all compounds, the pharmacokinetic parameters of AUC (0–*t*) and AUC (0–∞) values were increased. The study results may indicate that honey-processed FF slow these compounds elimination rate and enhance the bioavailability of these compounds. We speculate that the enhancement of moistening lung for arresting cough after honey-processed FF may be related to these active compounds. This conjecture is consistent with previous reports [6]. Standardized processing of FF as important as authentication to control their qualities and improve the curative effect.

In short, we can conclude that these eight active compounds can be quickly absorbed and play a pharmacodynamic effect in vivo. The results also proved the feasibility of honey-processed FF improving the curative effect of R-FF.

## 4. Conclusions

A fast, accurate UHPLC-MS/MS method was first established and validated for simultaneous determination of 8 analytes of R-FF and H-FF in rat serum. And, for all we know, this is the first report of pharmacokinetic comparison study between R-FF and H-FF. The pharmacokinetic parameter analysis indicates that honey-processed FF may change the pharmacokinetic behavior of the compounds, which provides the theoretical basis for the difference of the pharmacological activity between the raw and honey-processed FF. These results may contribute to clarify relevant basic and clinical research studies and the honey-processed mechanism.

## Figures and Tables

**Table 1:** Optimized SRM parameters of 8 analytes and IS.

Peak no.	Analytes	Retention time (min)	Precursor ion ([M − H]^−^) (*m/z*)	Product ion (*m/z*)	Collision energy (V)	Polarity
1	Ferulic acid	13.05	193.00	134.00; 177.97	27.93; 13.03	Negative
2	Caffeic acid	6.11	178.96	107.0; 135.0	22.66; 15.53	Negative
3	Chlorogenic acid	5.65	353.04	126.92; 190.97	34.30; 16.75	Negative
4	Neochlorogenic acid	2.94	353.04	178.97; 190.97	18.23; 18.87	Negative
5	Cryptochlorogenic acid	6.14	353.04	173.04; 178.97	15.65; 16.26	Negative
6	Isochlorogenic acid B	12.86	515.00	173.05; 353.07	26.91; 18.98	Negative
7	Isochlorogenic acid C	14.40	515.10	172.97; 353.16	28.35; 17.92	Negative
8	Rutin	13.17	610.00	300.04; 301.01	36.35; 34.64	Negative
IS	Chloramphenicol	10.85	321.00	152.07; 257.07	16.26; 16.22	Negative

**Table 2:** The regression equations, linear range, and LLOQs of the 8 analytes.

Analytes	Range (ng/mL)	Calibration curves	Correlation coefficient (*r*^2^)	LLOQ (ng/mL)
Ferulic acid	2.05–1000.00	*y* = 0.027*x*−0.857	0.993	2.05
Caffeic acid	2.78–8840.00	*y* = 0.035*x*−14.394	0.999	2.78
Chlorogenic acid	4.35–118500.00	*y* = 0.002*x*−1.566	0.999	4.35
Neochlorogenic acid	2.67–39000.00	*y* = 0.013*x*−2.914	0.990	2.67
Cryptochlorogenic acid	2.38–168000.00	*y* = 0.032*x*−32.266	0.990	2.38
Isochlorogenic acid B	1.30–211000.00	*y* = 0.031*x*−2.370	0.991	1.30
Isochlorogenic acid C	4.87–230000.00	*y* = 0.057*x*−9.097	0.990	4.87
Rutin	1.45–8150.00	*y* = 0.020*x* + 1.045	0.994	1.45

**Table 3:** Intraday and interday accuracy and precision of 8 analytes.

Analytes	Spiked concentration (ng/ml)	Intraday (*n* = 6)		Interday (*n* = 6)	
		Accuracy (RE, %)	Precision (RSD, %)	Accuracy (RE, %)	Precision (RSD, %)
Ferulic acid	100.00	4.09	3.48	4.73	4.89
	200.00	1.42	1.12	3.79	3.92
	500.00	2.14	2.18	4.81	3.55

Caffeic acid	380.00	1.43	3.08	−5.11	3.57
	3800.00	4.34	3.63	4.31	3.27
	6000.00	−1.19	1.22	2.45	4.55

Chlorogenic acid	3500.00	−3.24	2.79	−4.54	3.75
	40400.00	−1.20	1.26	−3.56	3.78
	110000.00	−3.72	2.41	4.70	4.03

Neochlorogenic acid	1000.00	−3.15	3.61	4.37	4.51
	15000.00	1.07	2.83	2.00	2.64
	25000.00	1.06	1.85	−3.78	4.30

Cryptochlorogenic acid	2000.00	2.63	3.65	4.84	3.82
	50000.00	1.28	1.95	2.65	2.18
	100000.00	2.47	2.93	3.99	2.44

Isochlorogenic acid B	2000.00	1.56	1.84	3.90	3.51
	80000.00	−2.30	2.47	−2.24	4.79
	160000.00	1.19	2.33	2.39	3.47

Isochlorogenic acid C	1500.00	1.53	3.92	4.09	4.65
	7500.00	−2.45	2.88	4.23	3.02
	140000.00	−1.42	2.72	4.46	4.94

Rutin	600.00	1.78	3.98	3.85	3.00
	4000.00	2.41	2.64	4.38	2.10
	8000.00	1.04	2.35	2.87	4.81

Chloramphenicol (IS)	100	1.11	1.26	3.06	3.48

**Table 4:** Matrix effects and extraction recoveries for the analytes in rat plasma.

Analytes	Concentration (ng/mL)	Matrix effect (%, mean ± SD, *n* = 6)	Recovery (%, mean ± SD, *n* = 6)
Ferulic acid	100.00	91.70 ± 3.26	102.70 ± 4.97
	200.00	88.68 ± 2.80	100.49 ± 2.41
	500.00	98.99 ± 5.31	99.04 ± 4.10

Caffeic acid	380.00	81.94 ± 3.24	104.48 ± 5.80
	3800.00	85.03 ± 9.17	81.54 ± 3.06
	6000.00	95.54 ± 6.83	95.55 ± 7.83

Chlorogenic acid	3500.00	91.35 ± 4.79	100.07 ± 5.27
	40400.00	99.34 ± 7.87	99.48 ± 3.17
	110000.00	95.09 ± 4.47	95.81 ± 2.29

Neochlorogenic acid	1000.00	95.75 ± 4.72	90.14 ± 4.73
	15000.00	99.46 ± 8.90	99.29 ± 5.83
	25000.00	99.63 ± 3.71	99.28 ± 3.32

Cryptochlorogenic acid	2000.00	97.98 ± 2.49	97.94 ± 6.42
	50000.00	101.71 ± 3.20	99.30 ± 7.60
	100000	100.32 ± 6.42	97.65 ± 4.42

Isochlorogenic acid B	2000.00	101.26 ± 6.32	99.13 ± 7.56
	80000.00	99.45 ± 12.51	99.83 ± 4.23
	160000.00	99.88 ± 4.49	103.60 ± 3.72

Isochlorogenic acid C	1500.00	103.02 ± 5.12	100.54 ± 3.38
	7500.00	99.03 ± 7.34	98.80 ± 4.49
	140000.00	100.43 ± 4.79	90.31 ± 5.51

Rutin	600.00	99.57 ± 4.76	102.72 ± 3.55
	4000.00	98.70 ± 3.57	100.86 ± 6.63
	8000.00	99.11 ± 4.95	101.40 ± 4.63

Chloramphenicol (IS)	100.00	90.77 ± 6.66	92.85 ± 4.67

**Table 5:** Stability of 8 analytes and IS in rat plasma (*n* = 3).

Analytes	Concentration (ng/mL)	Short-term (RE%)	Long-term (RE%)	Freeze-thaw (RE%)
Ferulic acid	100.00	2.02	2.16	4.78
	200.00	−0.51	3.51	1.65
	500.00	0.79	1.03	1.23

Caffeic acid	380.00	−3.53	−3.82	4.73
	3800.00	1.00	1.23	4.70
	6000.00	1.03	1.97	1.01

Chlorogenic acid	3500.00	0.12	−2.47	−1.34
	40400.00	1.78	−1.56	−1.72
	110000.00	−2.36	−2.94	−2.67

Neochlorogenic acid	1000.00	2.04	2.83	4.96
	15000.00	0.62	−4.71	2.35
	25000.00	1.47	2.60	−3.54

Cryptochlorogenic acid	2000.00	2.36	1.02	1.73
	50000.00	1.54	3.73	1.67
	100000	2.25	2.06	3.86

Isochlorogenic acid B	2000.00	−0.21	2.84	2.90
	80000.00	1.10	2.06	1.59
	160000.00	1.41	−2.26	−1.12

Isochlorogenic acid C	1500.00	1.31	4.77	2.78
	7500.00	−0.29	1.42	3.65
	140000.00	0.87	3.63	2.79

Rutin	600.00	−0.53	−4.09	−2.83
	4000.00	0.77	3.92	−1.23
	8000.00	1.23	3.30	2.31

Chloramphenicol (IS)	100	0.74	−2.22	−4.77

**Table 6:** Pharmacokinetic parameters of the 8 compounds in rats after oral administration of R-FF extracts (mean ± SD, *n* = 6).

Compound name	*C*_max_ (ng/mL)	*T*_max_ (h)	*t*_1/2_ (h)	AUC_0–*t*_ (ng/L)	AUC_0–∞_ (ng/L)	MRT_0–*t*_ (h)
Caffeic acid	4466.83 ± 310.88	0.33 ± 0.00	32.31 ± 17.62	20170.48 ± 405.49	35243.49 ± 9993.30	14.33 ± 0.25
Ferulic acid	252.591 ± 25.68	0.33 ± 0.00	5.07 ± 1.87	852.73 ± 29.44	2142.091 ± 195.59	4.21 ± 0.11
Chlorogenic acid	75585.07 ± 3061.79	0.33 ± 0.00	33.39 ± 3.36	167278.14 ± 21752.90	236184.38 ± 75589.19	10.62 ± 0.82
Cryptochlorogenic acid	67806.79 ± 3098.94	0.33 ± 0.00	21.85 ± 9.25	169993.99 ± 5978.78	242406.33 ± 49101.77	11.19 ± 0.53
Neochlorogenic acid	14852.03 ± 2370.78	0.33 ± 0.00	30.13 ± 6.85	56034.70 ± 4221.63	86956.297 ± 8336.51	12.53 ± 1.19
Isochlorogenic acidB	25184.90 ± 1591.85	0.33 ± 0.00	9.68 ± 1.36	193886.50 ± 6583.48	200747.374 ± 7208.02	10.91 ± 1.04
Isochlorogenic acid C	12916.78 ± 3117.99	0.33 ± 0.00	20.36 ± 5.22	110510.52 ± 9500.31	119513.50 ± 7705.49	14.32 ± 1.56
Rutin	7431.05 ± 446.28	0.33 ± 0.00	30.73 ± 14.04	100474.64 ± 9127.45	211030.24 ± 37905.16	18.91 ± 0.17

**Table 7:** Pharmacokinetic parameters of the 8 compounds in rats after oral administration of H-FF extracts (mean ± SD, *n* = 6).

Compound name	*C*_max_ (ng/mL)	*T*_max_ (h)	*t*_1/2_ (h)	AUC_0–*t*_ (ng/L)	AUC_0–∞_ (ng/L)	MRT_0–*t*_ (h)
Caffeic acid	4546.03 ± 234.68	0.33 ± 0.00	78.91 ± 26.02	24077.72 ± 2058.94	66775.53 ± 12172.05	12.74 ± 0.38
Ferulic acid	380.29 ± 5.89	0.33 ± 0.00	3.31 ± 0.68	1755.91 ± 224.17	2253.06 ± 441.77	3.33 ± 0.11
Chlorogenic acid	80426.52 ± 6354.93	0.33 ± 0.00	32.87 ± 4.87	280872.79 ± 10762.66	428350.92 ± 41590.65	11.10 ± 0.16
Cryptochlorogenic acid	97347.78 ± 1148.09	0.33 ± 0.00	17.57 ± 1.57	350674.18 ± 16655.08	401169.15 ± 21996.95	13.22 ± 0.39
Neochlorogenic acid	24733.36 ± 3224.91	0.33 ± 0.00	14.72 ± 3.88	95663.97 ± 6559.48	116684.574 ± 15910.07	10.87 ± 0.44
Isochlorogenic acid B	52292.60 ± 3007.78	0.33 ± 0.00	16.77 ± 5.09	388737.23 ± 24473.56	455375.91 ± 14598.23	15.19 ± 0.19
Isochlorogenic acid C	28183.80 ± 3739.61	0.33 ± 0.00	22.14 ± 9.73	186463.54 ± 18492.14	231840.44 ± 35996.24	16.42 ± 0.84
Rutin	7035.33 ± 429.34	0.39 ± 0.09	14.28 ± 2.28	64616.54 ± 4911.51	105151.44 ± 9984.91	22.73 ± 0.82

## Data Availability

The data used to support the findings of this study are included within the article.

## Supplementary Materials

Supplementary Materials The graphical abstract briefly summarizes the framework of this research experiment, which enables readers to quickly understand the thought, process, and the results of this experiment. Table S1 summarized all the results of matrix effect and extraction recoveries of eight compounds and internal standard compounds. Table S2 summarized the results of stability, including short-term stability, long-term stability, and freeze-thaw cycle.
