# Supplementary material for: Pharmacokinetic Comparisons of Eight Active Components from Raw Farfarae Flos and Honey-Processed Farfarae Flos after Oral Administration in Rats by UHPLC-MS/MS Approaches
Source: J Anal Methods Chem. 2020 May 20;2020:4091816. doi: 10.1155/2020/4091816 (PMC7256776; doi:10.1155/2020/4091816)
Supplement: Supplementary Materials — The graphical abstract briefly summarizes the framework of this research experiment, which enables readers to quickly understand the thought, process, and the results of this experiment. Table S1 summarized all the results of matrix effect and extraction recoveries of eight compounds and internal standard compounds. Table S2 summarized the results of stability, including short-term stability, long-term stability, and freeze-thaw cycle. [file 4091816.f1.zip › 4091816.f1/Annex II_JAMC_2920009.pdf]

Supplementary materials

Table S1. Matrix effects and extraction recoveries for the analytes in rat plasma

Analytes	Concentration (ng/mL)	Matrix effect (%, mean±SD, n=6)	Recovery (%, mean±SD, n=6)
Ferulic acid	100.00	91.70 ± 3.26	102.70 ± 4.97
	200.00	88.68 ± 2.80	100.49 ± 2.41
	500.00	98.99 ± 5.31	99.04 ± 4.10
Caffeic acid	380.00	81.94 ± 3.24	104.48 ± 5.80
	3800.00	85.03 ± 9.17	81.54 ± 3.06
	6000.00	95.54 ± 6.83	95.55 ± 7.83
Chlorogenic acid	3500.00	91.35 ± 4.79	100.07 ± 5.27
	40400.00	99.34 ± 7.87	99.48 ± 3.17
	110000.00	95.09 ± 4.47	95.81 ± 2.29
Neochlorogenic acid	1000.00	95.75 ± 4.72	90.14 ± 4.73
	15000.00	99.46 ± 8.90	99.29 ± 5.83
	25000.00	99.63 ± 3.71	99.28 ± 3.32
Cryptochlorogenic acid	2000.00	97.98 ± 2.49	97.94 ± 6.42
	50000.00	101.71 ± 3.20	99.30 ± 7.60
	100000	100.32 ± 6.42	97.65 ± 4.42
Isochlorogenic acid B	2000.00	101.26 ± 6.32	99.13 ± 7.56
	80000.00	99.45 ± 12.51	99.83 ± 4.23
	160000.00	99.88 ± 4.49	103.60 ± 3.72
Isochlorogenic acid C	1500.00	103.02 ± 5.12	100.54 ± 3.38
	7500.00	99.03 ± 7.34	98.80 ± 4.49
	140000.00	100.43 ± 4.79	90.31 ± 5.51
Rutin	600.00	99.57 ± 4.76	102.72 ± 3.55
	4000.00	98.70 ± 3.57	100.86 ± 6.63
	8000.00	99.11 ± 4.95	101.40 ± 4.63
Chloramphenicol (IS)	100.00	90.77 ± 6.66	92.85 ± 4.67

Table S2. Stability of 8 analytes and IS in rat plasma (n=3)

Analytes	Concentration (ng/mL)	Short-term (RE%)	Long-term (RE%)	Freeze-thaw (RE%)
Ferulic acid	100.00	2.02	2.16	4.78
	200.00	-0.51	3.51	1.65
	500.00	0.79	1.03	1.23
Caffeic acid	380.00	-3.53	-3.82	4.73
	3800.00	1.00	1.23	4.70
	6000.00	1.03	1.97	1.01
Chlorogenic acid	3500.00	0.12	-2.47	-1.34
	40400.00	1.78	-1.56	-1.72
	110000.00	-2.36	-2.94	-2.67
Neochlorogenic acid	1000.00	2.04	2.83	4.96
	15000.00	0.62	-4.71	2.35
	25000.00	1.47	2.60	-3.54
Cryptochlorogenic acid	2000.00	2.36	1.02	1.73
	50000.00	1.54	3.73	1.67
	100000	2.25	2.06	3.86
Isochlorogenic acid B	2000.00	-0.21	2.84	2.90
	80000.00	1.10	2.06	1.59
	160000.00	1.41	-2.26	-1.12
Isochlorogenic acid C	1500.00	1.31	4.77	2.78
	7500.00	-0.29	1.42	3.65
	140000.00	0.87	3.63	2.79
Rutin	600.00	-0.53	-4.09	-2.83
	4000.00	0.77	3.92	-1.23
	8000.00	1.23	3.30	2.31
Chloramphenicol (IS)	100	0.74	-2.22	-4.77
