# Supplementary material for: Pharmacokinetic Comparisons of Eight Active Components from Raw Farfarae Flos and Honey-Processed Farfarae Flos after Oral Administration in Rats by UHPLC-MS/MS Approaches
Source: J Anal Methods Chem. 2020 May 20;2020:4091816. doi: 10.1155/2020/4091816 (PMC7256776; doi:10.1155/2020/4091816)
Supplement: Supplementary Materials — The graphical abstract briefly summarizes the framework of this research experiment, which enables readers to quickly understand the thought, process, and the results of this experiment. Table S1 summarized all the results of matrix effect and extraction recoveries of eight compounds and internal standard compounds. Table S2 summarized the results of stability, including short-term stability, long-term stability, and freeze-thaw cycle. [file 4091816.f1.zip › 4091816.f1/Annex III_JAMC_2920010.pdf]

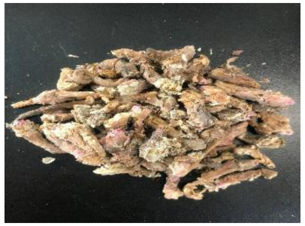

Raw Farfarae flos

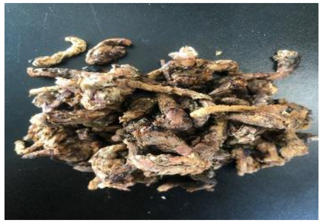

Honey-processed Farfarae flos

The plasma of Oral administration of raw, honey-processed Farfarae flos

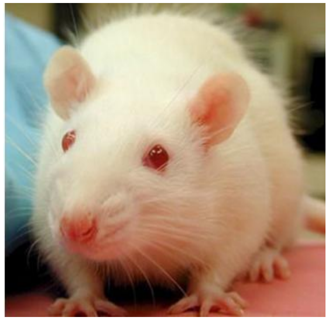

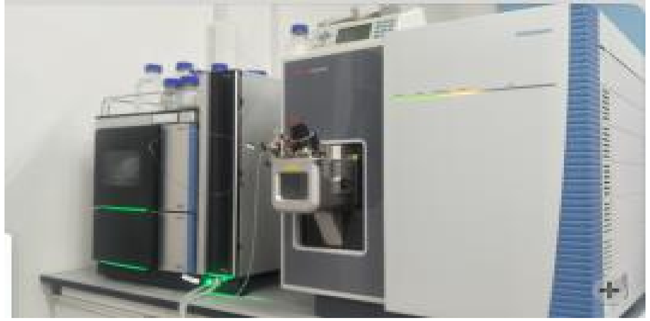

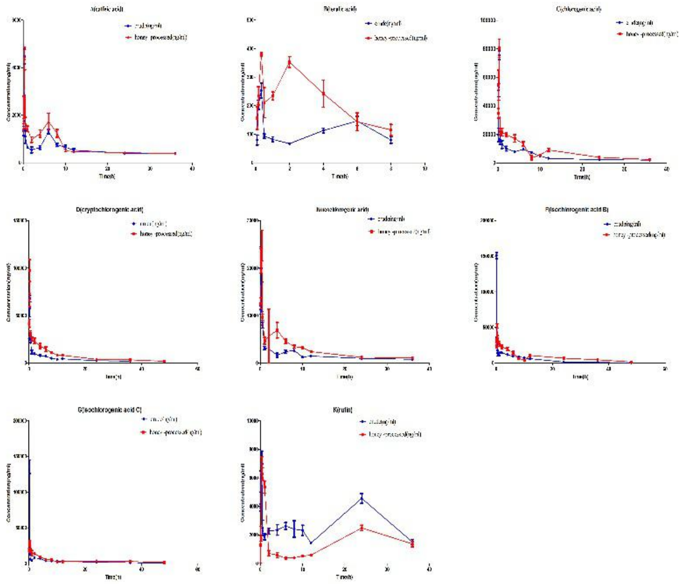

The mean plasma drug concentration–time curves
